# Multiparty Evolutionary Game Strategy for Green Technology Innovation Under Market Orientation and Pandemics

**DOI:** 10.3389/fpubh.2021.821172

**Published:** 2022-01-25

**Authors:** Min Gong, Aiming Dai

**Affiliations:** ^1^School of Management, Nanchang University, Nanchang, China; ^2^School of International Trade and Economics, Nanchang, China; ^3^School of International Trade, Jiangxi University of Finance and Economics, Nanchang, China

**Keywords:** pandemics, multiparty evolutionary game strategy, green technology innovation, market orientation, simulating theoretical analyses

## Abstract

Building a market-oriented green technology innovation system is important for China's green development. In this system, the government, enterprises, and consumers promote green innovation. Given this backdrop, this study constructs an evolutionary game model that combines the government, enterprises and consumers to analyse their evolution trend of strategy by simulating theoretical analyses. It is found that government subsidies for enterprises and consumers, benefits of enterprises speculation, and green consumption costs affect the enterprise decisions of green innovation. These factors significantly affect the enterprises' decision-making of green technology innovation. It is also observed that the market mechanism motivates enterprises' green technology innovation under pandemics. It is suggested that adopting more green consumption subsidy policies, improving the supervision mechanism and formulating more incentive policies from other aspects will be useful policy implications.

## Introduction

Green technology innovation also plays an important role in developing ecological civilisation construction. The report of the 19th Chinese Communist Party (CCP) National Congress indicates that to fulfil the green development concept of “two mountains,” persist in the basic state policy of saving resources and protecting the environment, develop a green way of development and a green way of life, cultivate the green product consumption concept. Meanwhile, the report also proposed to build a market-oriented system of green technology innovation. Market orientation of green technology innovation system refers to the specific network composed of relevant factors in green technology, based on the main market part, market rules, market system, market mechanism, to promote green technology research and development, diffusion and applications.

Expanding the scale of green product consumption and enhancing green product consumption is an effective way to improve ecological civilisation and green development. There is a constructional imbalance between lack of high-end product demand and excess supply of low-end in the green product market. On the supply side, the enterprises have less motivation to research and production green products, innovation ability and the core competitiveness is not strong, which leads to insufficient products supply; On the demand side, the price of a green product is too high to afford, the market demand remains to be further explored. Therefore, market orientation needs to solve the imbalance between green product supply and demand. Especially, enterprise's green technology innovation should be paid more attention. In this paper, a three-party evolutionary game model of government, enterprises and consumers is established to deeply analyse enterprises' green technology innovation behaviour under the guidance of the market.

Because of environmental pollution and ecological destruction, sustainable development has become the primary goal and strategy of social development. Under this background, firms began to change the traditional mode of production at the expense of the consumption of resources into making a green technology innovation strategy, guided by the scientific concept of development, to develop green products and a green market (1).

The issues of green technology innovation also gradually aroused the attention of many scholars. The research on this problem mainly focuses on the following aspects. Firstly, about the concept of green technology innovation. Scholars have defined green innovation from different perspectives, like product life cycle, environmental value, ecological benefit, and sustainable development (2). Different scholars' definitions of green innovation suggested that the concept of green innovation mainly covers six aspects, including innovation objects, market orientation, environmental aspects, phase, impulse, motivation, and level (3).

Secondly, scholars' studies mainly focus on green innovation's driving forces and influencing factors. In particular, the research of government policies on enterprises' green technology innovation has gained more attention. Because the enterprise green innovation behaviour has strong externality characteristics, the government's environmental policy for promoting green innovation is necessary. The government environmental policy can be mainly divided into two types: “market-oriented incentives policy” and “commanding regulation policy” (4). Through the comparative empirical analysis of two kinds of policy effects of green technology innovation, Jaffe et al. (5) proved that the incentive effect of market-oriented incentives environmental policy is more significant than that of commanding regulatory policies. However, He (6) observed that combining the Research and Development (R&D) subsidy policy and environmental regulation policy affects green technology R&D. The diffusion of enterprises in parallel by constructing a green technology innovation inducement mechanism model with the dual interaction between R&D subsidies and environmental regulatory policies.

Meanwhile, Li et al. (7) revealed the interactive process between the government and enterprises in green innovation activities by establishing an evolutionary game model between the government and enterprises under the joint mechanism of rewards, punishments and compensations. They analysed the evolutionary impact of innovation subsidy and failure compensation rates on the green innovation system. Shao et al. (8) studied the government subsidy policy in the new-energy automobile industry, divided the subsidy into two stages: R&D subsidy and production subsidy, and concluded that the utility of R&D subsidy was better than that of production subsidy.

Thirdly, because of increasing green demand, scholars' research perspective has gradually turned into the issues of enterprises' green innovation based on market-oriented. It elaborates on the scientific connotation and significance of building a market-orientated green technology innovation system and analyses the current problems in the green technology innovation system in china. Some suggestions were put forward to constructing a market-oriented green technology innovation system (9). De Medeiros et al. (10) provided the driving factors for the success of green innovation in the market, like consumer expectation and consumer behaviour variables were both key indicators of the internal relationship between innovation performance and green consumption preference. The authors proposed that the mechanism of price and competition, the allocation of innovation factors could be further optimised to promote green technology innovation of enterprises and described the influence path of market mechanism on enterprises' green technology innovation. In general, there are still few research results on green innovation from the perspective of market orientation, and this issue should be further studied in future.

From the above analysis, some research achievements have been made in green enterprise innovation, relations between government regulation and green innovation, market-orientated green innovation, and green consumption. However, the existing research has some insufficiencies. The research objects of most kinds of literature are single, and there is a lack of systematic analysis combing with all the participants in green innovation activities (11). Although the enterprise is the main body in the green technology innovation system, green technology innovation should be promoted through the integration, penetration and interaction between different system elements. Therefore, it is necessary to systematically analyse the issues of green technology innovation. This paper will analyse the influencing factors of enterprises' green innovation decision-making based on market orientation by constructing a three-party evolutionary game model of government, enterprises and consumers.

The rest of the paper is organised as follows. Section 2 explains the green innovation by a model construction. Section 3 provides the evolutionary game equilibrium analysis, and section 4 concludes.

## Green Innovation and Model Construction

**Figure 1:**
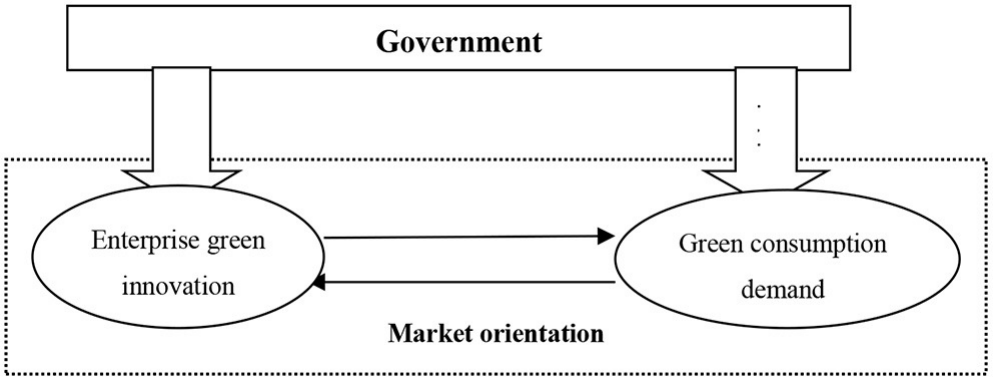
The relationships among the government, enterprises and consumers under market orientation.

Market orientation is an important driving force for enterprises' green technology innovation. In the face of consumers' demand for green products, enterprises may not necessarily invest in green product innovation because the cost is too high. So, there should be a mutual game between enterprises and consumers. At the same time, the government subsidy policy also has a certain impact on enterprises' green innovation-decision and consumers' purchasing decision-making. The relations are shown in Figure 1.

Therefore, this paper selects the government, enterprises and consumers as participants from the perspective of market demand and assumes that all three parties meet bounded rationality and make optimal decisions. The strategies of the three parties are government (subsidy, no subsidy); enterprise (innovation, no innovation); consumers (buy, do not buy). Suppose the government implements green innovation subsidies for enterprises and green consumption subsidies for consumers. When enterprises invest in green technology innovation, they will supply green products to the market. Nevertheless, no green innovation strategy means that they will still supply ordinary products produced to the market without green innovation. Consumers' purchase decision refers to the buying green products provided by enterprises in their green innovation. In contrast, the not-buying decision refers to the decision of consumers to buy traditional products instead of green products. The variables are assumed as follows.

### The Government

*g*_1_ is the basic benefit of the government. Assuming enterprises do not carry out green technology innovation, the government still obtains basic benefits, mainly from tax revenue. Δ*g*_1_ is the government's potential benefits brought by enterprises' green technology innovation, consuming green products, and developing the industry because green technology innovation can increase the optimisation of the social environment. If consumers do not buy green products, then Δ*g*_1_ = 0. *s*_1_ is government R&D subsidies to encourage enterprises to carry out green technology innovation and *s*_2_ consume subsidies to encourage consumers to carry out green consumption. If enterprises do not make any green innovation, consumers have to buy ordinary products, then, *s*_1_ = 0 and *s*_2_ = 0.

Suppose that the enterprise does not make any green technology innovation. In that case, they will adopt traditional technology for production, which will bring certain pollution and energy consumption to the society, the government will have extra cost on environmental governance. In this case, *p*_1_ is assumed to be the governance cost on environment pollution caused by producing in traditional non-green innovation technology, and *p*_2_ is assumed to be the governance cost on environment pollution when consumers purchase ordinary products. The government's strategy is subsidy and no subsidy, and the probabilities are, respectively, *x* and 1 − *x*, where 0 < *x* < 1.

### Enterprises

π_1_ is the basic income of the enterprise without green technology innovation. That is, the enterprise sells ordinary products produced by traditional technology. It can also be regarded as the opportunity cost of green technology innovation because enterprise innovation can be saved. However, in some cases, because of consumer preference for green products, the enterprise may deceive consumers and falsely claim they are selling green products to gain extra profits. Instead, they can change product packaging or increase advertising investment to gain the consumers' trust.

Δπ_1_ is assumed to be the revenue increment brought by the increase of sales after the implementation of green technology innovation, but if consumers do not choose green products, then Δπ_1_ = 0. $\Delta {\pi_{1}}^{{}^{\prime }}$ is the potential benefits obtained by enterprises under government subsidies for green innovation. Due to the green technology innovation, enterprises' market power and brand influence should be improved, and overall economic and social benefits will increase. *c*_1_ is the production cost of ordinary products without green technology innovation, Δ*c*_1_ which is the cost increment generated by green products under green technology innovation. The probabilities of innovation and non-innovation are *y* and 1 − *y*, respectively, where 0 < *y* < 1.

### Consumers

π_2_ is the expected utility of consumers who buy ordinary products instead of green products ${\pi_{2}}^{{}^{\prime }}$ is the expected utility of purchasing green products. The expected unity of consumers mainly refers to the difference between the use-value and the purchase cost of the product. We assumed that ${\pi_{2}}^{{}^{\prime }}>$π_2_ if the enterprise does not innovate green technology, the potential loss when consumers purchase fake or low-tech “green products” can be an opportunity cost *c*_2_. The consumer's strategy is to buy or not to buy, and the probabilities are *z* and 1 − *z*, respectively, where 0 < *z* < 1.

**Figure 2:**
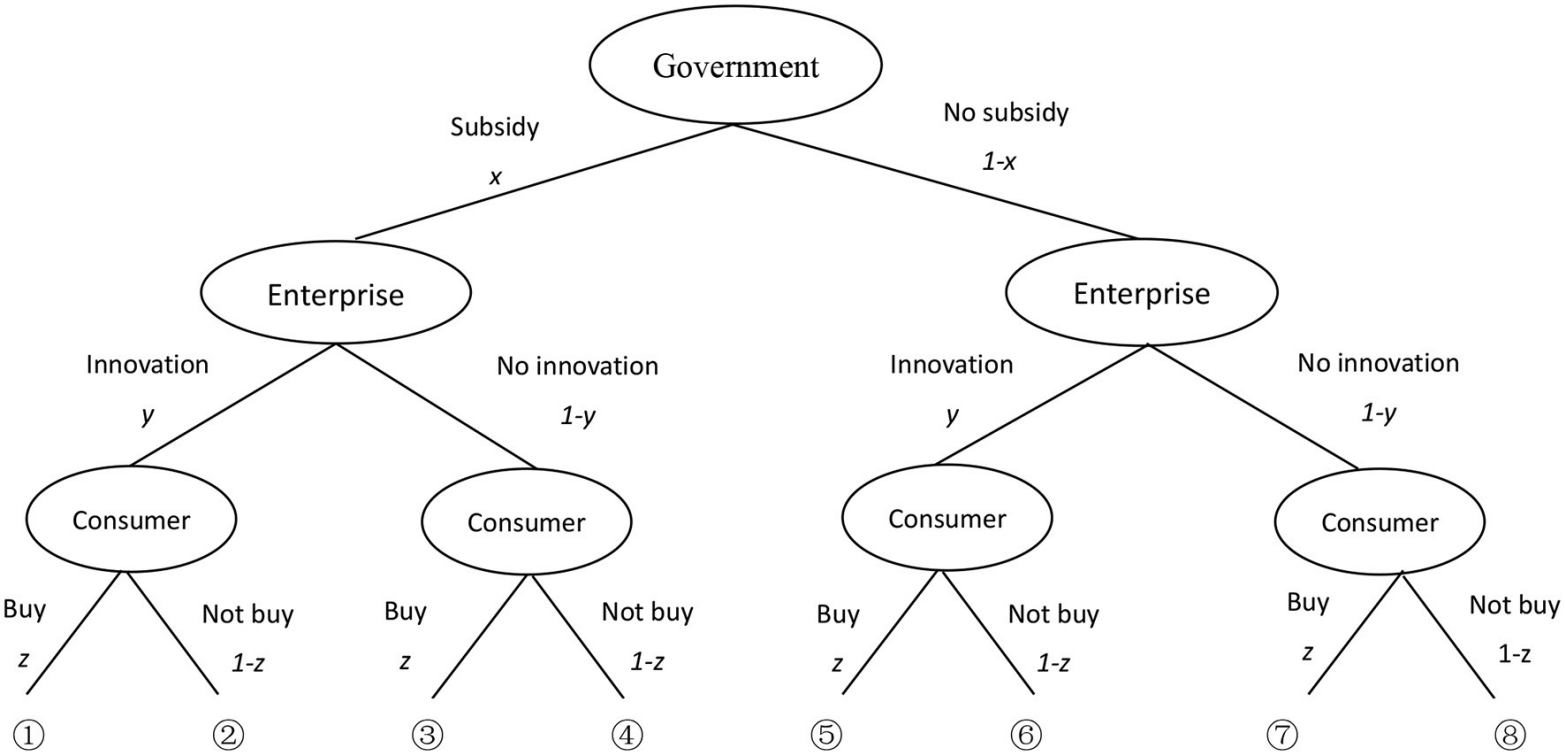
Tripartite game model of government, enterprises and consumers.

Based on the above assumptions of profit and loss variables, the game tree is constructed, as shown in Figure 2.

According to the decision tree model of the three parties in Figure 2 above, there are eight combined options for the government, enterprises, and consumers. The income matrix is obtained as shown in Table 1.

**Table 1:** Revenue matrix of government, enterprises and consumers.

**Options**	**The set of actor strategies**	**Three parties' benefit**
1	(Subsidy, innovation, buy)	*g*_1_+Δ*g*_1_ − *s*_1_ − *s*_2_ $\pi_{1}+\Delta \pi_{1}+\Delta {\pi_{1}}^{{}^{\prime }}+s_{1}-c_{1}-\Delta c_{1}$ $\pi_{2}^{{}^{\prime }}+s_{2}$
2	(Subsidy, innovation, not buy)	*g*_1_ − *s*_1_ − *p*_2_ $\pi_{1}+\Delta {\pi_{1}}^{{}^{\prime }}+s_{1}-c_{1}-\Delta c_{1}$ π_2_
3	(Subsidy, no innovation, buy)	*g*_1_ − *p*_1_ − *p*_2_ $\pi_{1}+{\pi_{1}}^{{}^{\prime }}-c_{1}$ $s_{2}+{\pi_{2}}^{{}^{\prime }}-c_{2}$
4	(Subsidy, no innovation, not buy)	*g*_1_ − *p*_1_ − *p*_2_ π_1_ − *c*_1_ ${\pi_{2}}^{{}^{\prime }}$
5	(No subsidy, innovation, buy)	*g*_1_+Δ*g*_1_ $\pi_{1}+\Delta {\pi_{1}}^{{}^{\prime }}-c_{1}-\Delta c_{1}$ ${\pi_{2}}^{{}^{\prime }}$
6	(No subsidy, innovation, not buy)	*g*_1_ − *p*_2_ π_1_ − *c*_1_ − Δ*c*_1_ π_2_
7	(No subsidy, no innovation, buy)	*g*_1_ − *p*_1_ − *p*_2_ $\pi_{1}+{\pi_{1}}^{{}^{\prime }}-c_{1}$ ${\pi_{2}}^{{}^{\prime }}-c_{2}$
8	(No subsidy, no innovation, not buy)	*g*_1_ − *p*_1_ − *p*_2_ π_1_ − *c*_1_ π_2_

## Evolutionary Game Equilibrium Analysis

Expected revenue analysis of the three parties: According to the revenue matrix in Table 1, the expected revenue of the three parties of government, enterprise and consumer can be obtained as follows:

Expected revenue of the government: Suppose that the government's expected revenue is *U*_11_ when the subsidy policy is implemented and is the government's expected revenue when the subsidy policy is not implemented. The average expected return of the government is ${\bar{U}}_{1}$, ${\bar{U}}_{1}=xU_{11}+\left(1-x\right)U_{12}$. Calculate *U*_11_ and *U*_12_:


(1a) $$\begin{array}{l} \begin{array}{l} U_{11}=yz\left(g_{1}+\Delta g_{1}-s_{1}-s_{2}\right)+y\left(1-z\right)\left(g_{1}-s_{1}-p_{2}\right)\\ \;+\left(1-y\right)z\left(g_{1}-p_{1}-p_{2}\right)+\left(1-y\right)\left(1-z\right)\\ \;\left(g_{1}-p_{1}-p_{2}\right)=yz\Delta g_{1}-yzs_{2}+yzp_{2}-ys_{1}+yp_{1}\\ \;+g_{1}-p_{1}-p \end{array} \end{array}$$

(1b) $$\begin{array}{l} \begin{array}{l} U_{12}=yz\left(g_{1}+\Delta g_{1}\right)+y\left(1-z\right)\left(g_{1}-p_{2}\right)\\ \;+\left(1-y\right)z\left(g_{1}-p_{1}-p_{2}\right)+\left(1-y\right)\left(1-z\right)\\ \;\left(g_{1}-p_{1}-p_{2}\right)=yz\Delta g_{1}+yzp_{2}+yp_{1}\\ \;+g_{1}-p_{1}-p \end{array} \end{array}$$

Expected revenue of the enterprise: Suppose that the expected revenue of the enterprise is *U*_11_ when they have green technology innovation; if not, the expected revenue is, and the average expected revenue of the enterprise is ${\bar{U}}_{2}$. Then, ${\bar{U}}_{2}=yU_{21}+\left(1-y\right)U_{22}$. Calculate *U*_21_ and *U*_22_:


(2) $$\begin{array}{l} \begin{array}{l} U_{21}=xz\left(\pi_{1}+\Delta \pi_{1}+\Delta {\pi_{1}}^{{}^{\prime }}+s_{1}-c_{1}-\Delta c_{1}\right)\\ \;+x\left(1-z\right)\left(\pi_{1}+\Delta {\pi_{1}}^{{}^{\prime }}+s_{1}-c_{1}-\Delta c_{1}\right)\\ \;+\left(1-x\right)z\left(\pi_{1}+\Delta \pi_{1}-c_{1}-\Delta c_{1}\right)\\ \;+\left(1-x\right)\left(1-z\right)\left(\pi_{1}-c_{1}-\Delta c_{1}\right)\\ \;=x\Delta {\pi_{1}}^{{}^{\prime }}+xs_{1}+\pi_{1}+z\Delta \pi_{1}-c_{1}-\Delta c_{1} \end{array} \end{array}$$

(3) $$\begin{array}{l} U_{22}=xz\left(\pi_{1}+\Delta {\pi_{1}}^{{}^{\prime }}-c_{1}\right)+x\left(1-z\right)\left(\pi_{1}-c_{1}\right)\\ +\left(1-x\right)z\left(\pi_{1}+{\pi_{1}}^{{}^{\prime }}-c_{1}\right)+\left(1-x\right)\left(1-z\right)\left(\pi_{1}-c_{1}\right)\\ =z\Delta {\pi_{1}}^{{}^{\prime }}+\pi_{1}-c_{1} \end{array}$$

Expected revenue of the customer: Suppose *U*_31_ is the expected revenue when customers choose to buy green products instead of ordinary products, while *U*_32_ is the expected revenue when they do not buy green products. Then, ${\bar{U}}_{3}=zU_{31}+\left(1-z\right)U_{32}$. Calculate *U*_31_ and *U*_32_.


(4) $$\begin{array}{l} U_{31}=xy\left({\pi_{2}}^{{}^{\prime }}+s_{2}\right)+x\left(1-y\right)\left({\pi_{2}}^{{}^{\prime }}+s_{2}-c_{2}\right)+\left(1-x\right)y\left({\pi_{1}}^{{}^{\prime }}\right)+\\ \;\left(1-x\right)\left(1-y\right)\left(\pi_{2}^{{}^{\prime }}-c_{2}\right)=xs_{2}+\pi_{2}^{{}^{\prime }}-c_{2}+yc_{2}\\ U_{32}=xy\pi_{2}+x\left(1-y\right)\pi_{2}+\left(1-x\right)y\pi_{1}+\left(1-x\right)\left(1-y\right)\pi_{2}\\ \;=\pi_{2} \end{array}$$

### Dynamic Replication Equation of Tripartite Game

According to the tripartite revenue function, construct the game dynamic replication equation:


(5) $$\begin{array}{l} \{F(x)\text{ = }\frac{\partial x}{\partial t}=x(U_{11}-\overset{-}{U_{1}})=x(1-x)(yzs_{2}-ys_{1})=0\\ F(y)=\frac{\partial x}{\partial t}\;=y(U_{21}-\overset{-}{U_{2}})=y(1-y)(x\Delta \pi_{1}+xs_{1}+\pi_{1}\\ +z\Delta \pi_{1}-\Delta c_{1})=0\\ F(z)=\;\frac{\partial x}{\partial t}=z(U_{31}-\overset{-}{U_{3}})=z(1-z)(xs_{2}+\pi_{2}-c_{2}\\ +yc_{2}-\pi_{2})=0\} \end{array}$$

Solving the above equation, we can get nine equilibrium points: (0,0,0); (0,01,); (0,1,0); (0,1,1); (1,0,0); (1,0,1); (1,1,0); (1,1,1); (x^*^, y^*^, z^*^).


(6) $$\begin{array}{l} \{\begin{array}{l} x^{*}=\frac{\Delta c_{1}s_{2}-\Delta \pi_{1}+\pi_{1}}{s_{2}\left(\pi_{1}+s_{1}\right)}\\ y^{*}\text{=}\frac{\left(\pi_{2}-\pi_{2}+c_{2}\right)\left(\pi_{1}+s_{1}\right)-\left(\Delta c_{1}s_{2}-\Delta \pi_{1}+\pi_{1}\right)}{\pi_{1}+s_{1}}\\ z^{*}\text{=}\frac{s_{1}}{s_{2}} \end{array} \end{array}$$

Analysis of tripartite dynamic equilibrium: Because there is information asymmetry in the market, in the long term, the government, enterprises and consumers will adjust their strategies based on other subjects' decisions, so the game among the three parties has dynamic characteristics. Therefore, to determine the stability strategy of the system, we can establish a Jacobi matrix, judging by the stability criterion of the Jacobi matrix. J is for the Jacobi matrix.


(7) $$\begin{array}{l} \begin{array}{l} J=\left(\begin{array}{l} \frac{\partial F\left(x\right)}{\partial x}\frac{\partial F\left(x\right)}{\partial y}\frac{\partial F\left(x\right)}{\partial z}\\ \frac{\partial F\left(y\right)}{\partial x}\frac{\partial F\left(y\right)}{\partial y}\frac{\partial F\left(y\right)}{\partial z}\\ \frac{\partial F\left(z\right)}{\partial x}\frac{\partial F\left(z\right)}{\partial y}\frac{\partial F\left(z\right)}{\partial z} \end{array}\right)\\ =\left(\begin{array}{ccc} \left(1-2x\right)\left(yzs_{2}-ys_{1}\right) & x\left(1-x\right)\left(zs_{2}-s_{1}\right) & x\left(1-x\right)\left(ys_{2}\right)\\ y\left(1-y\right)\left(\Delta \pi_{1}+s_{1}\right) & \left(1-2y\right)\left(x\Delta \pi_{1}+xs_{1}+z\Delta \pi_{1}-z\pi_{1}-\Delta c_{1}\right) & y\left(1-y\right)\left(\Delta \pi_{1}-\pi_{1}\right)\\ z\left(1-z\right)s_{2} & z\left(1-z\right)c_{2} & \left(1-2z\right)\left(xs_{2}+\pi_{2}-c_{2}+yc_{2}-\pi_{2}\right) \end{array}\right) \end{array} \end{array}$$

According to evolutionary game theory, a Nash equilibrium is the system's dynamic balance, the evolution equilibrium must be a Nash equilibrium, and the equilibrium state must be pure strategy equilibrium (12). So in the analysis of the asymptotic stability of the system, the hybrid strategy could be excluded. Therefore, we just need to analyse combining the following eight equilibrium points. *E*_1_ (0,0,0), *E*_2_ (0,0,1), *E*_3_ (0,1,0), *E*_4_ (0,1,1), *E*_5_ (1,0,0), *E*_6_ (1,0,1), *E*_7_ (1,1,0), *E*_8_ (1,1,1).

Then, plug the eight equilibrium points into the matrix. According to Liapunov's stability criterion, the equilibrium point satisfying all the eigenvalues of the matrix are non-positive is the evolutionarily stable strategy (ESS), which is the dynamic equilibrium point of the system.

From Table 2, it can be seen that when the values of equilibrium points *E*_1_ (0,0,0), *E*_2_ (0,0,1), *E*_5_ (1,0,0), *E*_6_ (1,0,1) are substituted into the matrix, the eigenvalues are identically vanishing. There is no asymptotically stable point in the system at these equilibrium points. Therefore, we need to determine the values of eigenvalues at the equilibrium points *E*_3_ (0,1,0), *E*_4_ (0,1,1), *E*_7_ (1,1,0), *E*_8_ (1,1,1). Among them, the eigenvalues at the point *E*_3_ and *E*_7_ are positive, and the system does not have asymptotic stability. So we only need to discuss the eigenvalues at the point of *E*_4_ (0,1,1) and *E*_8_ (1,1,1). According to the above hypothesis $\pi_{2}^{{}^{\prime }}>\pi_{2}$, we can get $-\left(\pi_{2}^{{}^{\prime }}-\pi_{2}\right)<0$. The asymptotic stability point can be determined by the values of *s*_2_ − *s*_1_ and $\Delta \pi_{1}-\pi_{1}^{{}^{\prime }}-\Delta c_{1}$. Hypothesis analysis of relevant variables is discussed in the following four cases in Tables 3–5.

**Table 2:** Eigenvalues of the matrix corresponding to equilibrium points.

**Equilibrium points**	**Eigen value**	**Eigen value**	**Eigen value**
	**λ_**1**_**	**λ_**2**_**	**λ_**3**_**
*E*_1_(0,0,0)	0	− Δ*c*_1_	$\pi_{2}^{{}^{\prime }}-c_{2}-\pi_{2}$
*E*_2_(0,0,1)	0	$\Delta \pi_{1}-\pi_{1}^{{}^{\prime }}-\Delta c_{1}$	$-\left(\pi_{2}^{{}^{\prime }}-c_{2}-\pi_{2}\right)$
*E*_3_(0,1,0)	− *s*_1_	Δ*c*_1_	$\pi_{2}^{{}^{\prime }}-\pi_{2}$
*E*_4_(0,1,1)	*s*_2_ − *s*_1_	$-\left(\Delta \pi_{1}-\pi_{1}^{{}^{\prime }}-\Delta c_{1}\right)$	$-\left(\pi_{2}^{{}^{\prime }}-\pi_{2}\right)$
*E*_5_(1,0,0)	0	$\Delta {\pi_{1}}^{{}^{\prime }}+s_{1}-\Delta c_{1}$	$s_{2}+\pi_{2}^{{}^{\prime }}-c_{2}-\pi_{2}$
*E*_6_(1,0,1)	0	$\Delta {\pi_{1}}^{{}^{\prime }}+s_{1}+\Delta \pi_{1}-{\pi_{1}}^{{}^{\prime }}-\Delta c_{1}$	$-\left(s_{2}+\pi_{2}^{{}^{\prime }}-c_{2}-\pi_{2}\right)$
*E*_7_(1,1,0)	*s*_1_	$-\left(\Delta {\pi_{1}}^{{}^{\prime }}+s_{1}-\Delta c_{1}\right)$	$s_{2}+\pi_{2}^{{}^{\prime }}-\pi_{2}$
*E*_8_(1,1,1)	− (*s*_2_ − *s*_1_)	$-\left(\Delta {\pi_{1}}^{{}^{\prime }}+s_{1}+\Delta \pi_{1}-{\pi_{1}}^{{}^{\prime }}-\Delta c_{1}\right)$	$-\left(s_{2}+\pi_{2}^{{}^{\prime }}-\pi_{2}\right)$

**Table 3:** Case 1.

	**Eigen value**	**Eigen value**	**Eigen value**	**Stability judge**
		**λ_**1**_**	**λ_**2**_**	**λ_**3**_**
*E*_4_(0,1,1)	>0	<0	<0	Saddle point
*E*_8_(1,1,1)	<0	<0	<0	ESS

**Table 4:** Case 2.

	**Eigen value**	**Eigen value**	**Eigen value**	**Stability judge**
		**λ_**1**_**	**λ_**2**_**	**λ_**3**_**
*E*_4_(0,1,1)	>0	>0	<0	Saddle point
*E*_8_(1,1,1)	<0	<0	<0	ESS

**Table 5:** Case 3.

	**Eigen value**	**Eigen value**	**Eigen value**	**Stability judge**
		**λ_**1**_**	**λ_**2**_**	**λ_**3**_**
*E*_4_(0,1,1)	<0	<0	<0	ESS
*E*_8_(1,1,1)	>0	<0	<0	Saddle point

Suppose *s*_2_ − *s*_1_ > 0 and $\Delta \pi_{1}-\pi_{1}^{{}^{\prime }}-\Delta c_{1}>0$, then *E*_8_ (1,1,1) is the asymptotic stability point of the system.

- Suppose *s*_2_ − *s*_1_ > 0 and $\Delta \pi_{1}-\pi_{1}^{{}^{\prime }}-\Delta c_{1}>0$, then *E*_8_ (1,1,1) is the asymptotic stability point of the system.
- Suppose *s*_2_ − *s*_1_ > 0 and $\Delta \pi_{1}-\pi_{1}^{{}^{\prime }}-\Delta c_{1}<0$, then $\Delta {\pi_{1}}^{{}^{\prime }}+s_{1}+\Delta \pi_{1}-{\pi_{1}}^{{}^{\prime }}-\Delta c_{1}>0$ , at this time, *E*_8_ (1,1,1) is the asymptotic stability point of the system.
- Suppose *s*_2_ − *s*_1_ < 0 and $\Delta \pi_{1}-\pi_{1}^{{}^{\prime }}-\Delta c_{1}>0$, then *E*_4_ (0,1,1) is the asymptotic stability point of the system.
- Suppose *s*_2_ − *s*_1_ < 0 and $\Delta \pi_{1}-\pi_{1}^{{}^{\prime }}-\Delta c_{1}<0$, then there is no asymptotic stability point of the system.

In summary, if *s*_2_ − *s*_1_ > 0, then, $\Delta \pi_{1}-\pi_{1}^{{}^{\prime }}-\Delta c_{1}>0$ or $\Delta \pi_{1}-\pi_{1}^{{}^{\prime }}-\Delta c_{1}<0$ $\Delta {\pi_{1}}^{{}^{\prime }}+s_{1}+\Delta \pi_{1}-{\pi_{1}}^{{}^{\prime }}-\Delta c_{1}>0$, *E*_8_ (1,1,1) is the asymptotic stability point of the system. On the contrary, when *s*_2_ − *s*_1_ < 0 *E*_4_ (0,1,1) is the asymptotic stability point of the system. So we will analyse the dynamic evolution feature at the point of *E*_8_ (1,1,1) to simulate the three parties' dynamic evolution trend at a certain time.

### Simulation Analyses

Based on the dynamic replication equation and equilibrium analysis above, we simulate the dynamic evolution of the game strategy of the three parties. By setting parameters, analyse the influence of key parameters on the strategy of all the parties.

The initial value of the main parameters is set as: *x* = 0.4, *y* = 0.3, *z* = 0.2, and the other parameters are set as


$$\begin{array}{l} s_{1}=0.3,s_{2}=0.4,\Delta \pi_{1}^{{}^{\prime }}=0.3,\Delta \pi_{1}=0.7,\pi_{1}^{{}^{\prime }}=0.2,\\ \Delta c_{1}=0.2,c_{2}=0.3,\pi_{2}=0.5,\pi_{2}^{{}^{\prime }}=0.7. \end{array}$$

**Figure 3:**
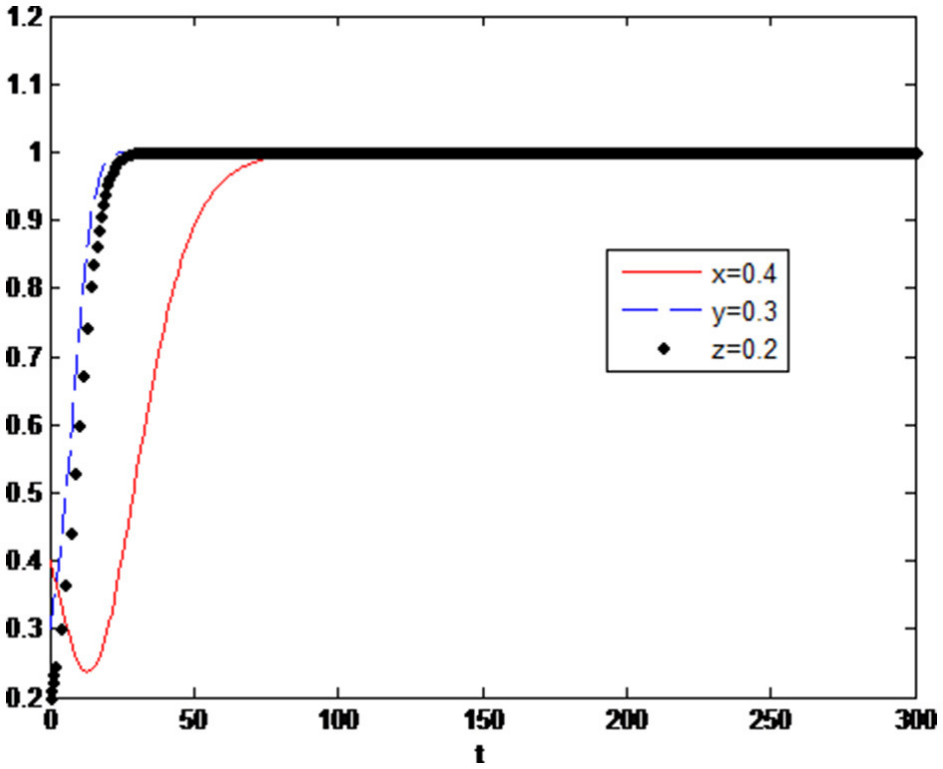
Three-party dynamic evolution simulation with an initial value setting.

Initial time *t* = 0 and the end time of evolution is *t* = 300. Simulation results are shown in Figure 3.

As shown in Figure 3, the government, enterprises and consumers are gradually making an optimal decision over some time. The system tends to the equilibrium point (1,1,1). The government chooses to implement different levels of subsidy policy both on enterprises and consumers to encourage enterprises to make more green technology innovations and encourage consumers to have more green consumption. In the case of government subsidies and consumers' preference for green consumption, the increment of visible and invisible benefits of green innovation is larger than the increment of costs, so the optimal strategy of enterprises will eventually be having green technology innovation. After comparing the cost and benefit of green consumption, rational consumers will also decide when the benefit is greater than the cost. To analyse the impact of these valuables changes on their evolutionary decisions, relevant parameters of governments, firms and consumers will be adjusted in the following.

### Values of *s*_1_ and *s*_2_

**Figure 4:**
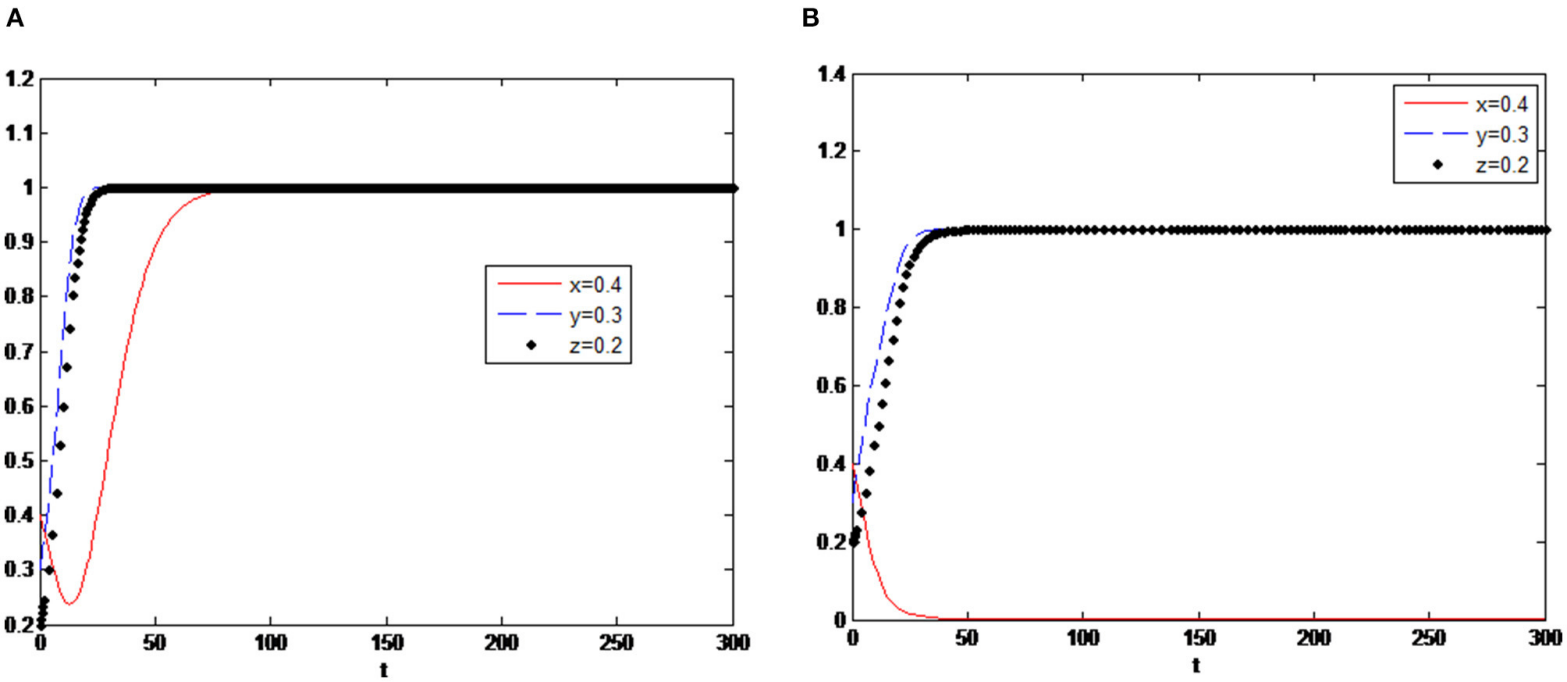
**(A)** Evolution simulation in case of *s*_1_ < *s*_2_. **(B)** Evolution simulation in case of *s*_1_ > *s*_2_.

In the first case, it is assumed that *s*_1_ < *s*_2_ as the initial setting, *s*_1_ = 0.3, *s*_2_ = 0.4, the simulation results are shown in Figure 4A, and the system tends to the stable point (1,1,1). However, the second case is assumed to be *s*_1_ > *s*_2_, set *s*_1_ = 0.4, *s*_2_ = 0.3, its simulation results in Figure 4B. The system tends to the unstable point (0,1,1). By comparing the result of the two cases, it proved that when the government's subsidies for green consumption are less than the innovation subsidies to enterprises, the government will give up the strategy of implementing subsidies, resulting in policy failure eventually. Because when green consumption subsidies are getting less, consumers' demand for green products will decrease.

On the other hand, high green innovation subsidies will make enterprises have a higher level of innovation and provide more green products, which will lead to market failure, that is, supply exceeds demand. So in this situation, the final decision of the three parties is (no subsidies, innovation, buy). Based on the result, it can be suggested that the government should consider the different subsidy levels for enterprises and consumers when implementing subsidy policies, and the system equilibrium is reached only when the consumption subsidy is greater than the innovation subsidy.

### Values Related to Enterprises

**Figure 5:**
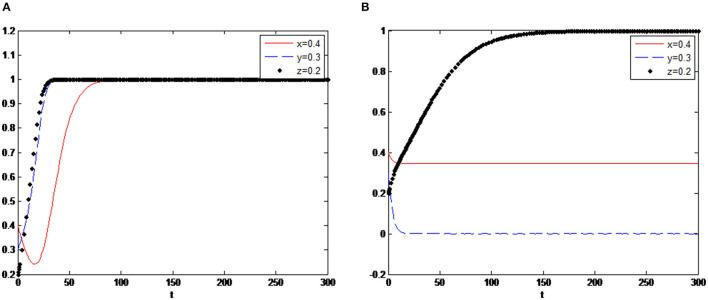
**(A)** Δπ_1_ = 0.8, Δ*c*_1_ = 0.5. **(B)** $\pi_{1}^{{}^{\prime }}=0.8$ Δ*c*_1_ = 0.5.

Assuming that other variables remain unchanged, adjust the values of enterprise variables, and the initial value setting Δπ_1_ = 0.7, Δ*c*_1_ = 0.2 is adjusted Δπ_1_ = 0.8, Δ*c*_1_ = 0.5. Then the system simulation results are shown in Figure 5A. It shows that neither the benefit increment of green innovation decrease nor cost increment increase will not have a big impact on the three parties' decision. The equilibrium point of the system is still (1,1,1). Although the innovation cost is crucial, there is still big market demand for green products to motivate the enterprise to innovate green technology.

Next, assume that adjust $\pi_{1}^{{}^{\prime }}=0.7$ to $\pi_{1}^{{}^{\prime }}=0.8$, Δ*c*_1_ = 0.2 to Δ*c*_1_ = 0.5. As can be seen from Figure 5B, values of $\pi_{1}^{{}^{\prime }}$ and Δ*c*_1_ have a certain influence on the evolution trend of the system. Deceiving consumers can bring much more extra profits (the opportunity cost of innovation), enterprises become the opportunist, in the long run, they will lose its motivation of green innovation, that is why many companies will falsely claim they have carried on the green innovation, not only deceive the consumers, and defraud the government for the green innovation subsidies. Under such circumstances, the government will take a cautious attitude toward the subsidy policy and exert its supervision function to prevent the speculative behaviour of enterprises.

**Figure 6:**
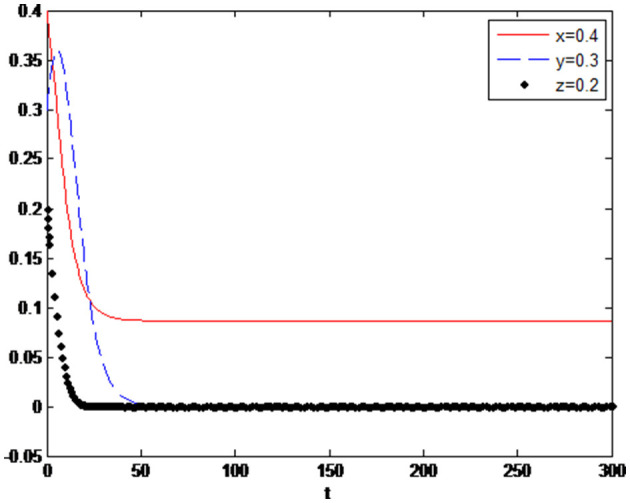
In case of *c*_2_ = 0.8, $\pi_{1}^{{}^{\prime }}=0.7$.

Values of consumer-related variables: Assuming the other variables remain unchanged, adjust the variables related to consumers, adjust the initial value *c*_2_ = 0.3 to *c*_2_ = 0.8, and the system simulation results are shown in Figure 6. It is shown that the system equilibrium tends to (0,0,0). In this case, considering the high cost of green consumption, consumers finally give up the consumption of green products, which will lead to low demand for green products. Eventually, the enterprise will lose the motivation to carry out green technology innovation, and the government will give up the subsidy policy due toineffectiveness.

## Conclusion

In the simulation process of the above model, adjustments were made to variables such as government subsidies, related benefits of enterprises and cost benefits of consumers. It was found that the adjustment of some variables would change the change of the equilibrium of the whole system, which means that these variables greatly influence the strategies of the government, enterprises, and consumers. According to the setting and analysis of the above parameters, the following conclusions are drawn: Firstly, the government's incentive effect of green consumption subsidies is more significant than green innovation subsidies for enterprises. The green consumption subsidy of the government stimulates and increases the demand of consumers for green products, thus further improving the sales profit of green products for enterprises and overall social welfare. When the government's subsidies for green consumption are greater than those for enterprises' green innovation, the three parties will evolve into a system equilibrium point. On the contrary, when consumption subsidies are less than the green innovation subsidies, the government's subsidies policy effect will not be obvious. Green innovation and green consumption cannot be effectively motivated.

Secondly, the opportunity cost of green innovation will influence enterprises' green innovation decisions. Due to the lack of supervision and punishment mechanism in the market, driven by profits, enterprises will take opportunistic behaviours or adverse selection in the process of green innovation, which will fail the market mechanism, and enterprises will eventually give up the decision of green technology innovation. Therefore, the government should strengthen the supervision and punishment of opportunistic behaviour of enterprises, and regulate market competition to prevent adverse selection or moral hazard behaviour, so that the market mechanism can be more effective.

Thirdly, green consumption costs significantly affect enterprises' green technology innovation decisions. From the above analysis, the high cost of green consumption will lead consumers to give up green consumption and make enterprises lose the motivation of green technology innovation. This conclusion reflects that market orientation is necessary for enterprises' green technology innovation. Under the market orientation, consumers' green demand has a reversed t in the transmission of pressure to make enterprises' have green technology innovation. By reducing the cost of green consumption, consumers' demand for green products can be stimulated to improve enterprises' motivation for green innovation.

Based on the above analysis, the following enlightenment can be drawn: market orientation is a necessary condition for enterprises to carry out green innovation; however, the role of the government in enterprises' green technology innovation with the market-oriented mechanism cannot be ignored, as it plays the role of “pushing hand” in coordinating and ensuring the green innovation activities (13).

To more effectively stimulate enterprises' green technology innovation, the government should implement the following policy implications given the significant impact of the COVID-19 pandemic. Firstly, adopt more green consumption subsidy policies to stimulate consumer demand for a green product, create a good market-oriented external environment, and form a benign interaction between green technology innovation, green consumption demand and industrial development. Secondly, the government and society should establish and improve the supervision mechanism, strengthen the supervision and punishment of opportunistic enterprise behaviour, prevent the occurrence of adverse behaviour and moral hazard, reduce the phenomenon of “market failure,” and make the market mechanism play a better guiding role in promoting green enterprise innovation. Thirdly, formulate more incentive policies from other aspects. The government should formulate various incentive policies to promote the development of the green product market to force enterprises to have green technology innovation. For example, on the supply side, through the formulation of a green financial policy system, enterprises can optimise the allocation of green technology innovation resources, reduce their innovation costs to lower the price of green products; On the demand side, the government should increase the publicity of green consumption and green products, to raise consumers' cognitive of green products and cultivate consumers' concept of green consumption.

By establishing an evolutionary game model among the government, enterprises, and consumers, this paper analyses the three parties' equilibrium strategy and uses the Matlab software for simulation. By adjusting the model's key variable parameters, we determine the key variables affecting consumers' green product consumption and enterprises' green innovation decision-making under the market orientation. Based on this, relevant policy suggestions are put forward to promote the driving effect of market mechanisms on the green technology innovation of enterprises. The deficiencies of this paper are as follows: firstly, to simplify the analysis, the variable assumption of consumer green product consumption is relatively simple. Only the cost and expected income of green consumption are generally considered; Secondly, although the key variables that determine the consumers' green consumption and enterprises' green innovation decisions have been found through evolutional simulation analysis, their influencing mechanism is not elaborated problem remains for future research.

## Data Availability Statement

The original contributions presented in the study are included in the article/supplementary material, further inquiries can be directed to the corresponding author/s.

## Author Contributions

MG: introduction and modelling. AD: literature review and conclusion. All authors contributed to the article and approved the submitted version.

## Conflict of Interest

The authors declare that the research was conducted in the absence of any commercial or financial relationships that could be construed as a potential conflict of interest.

## Publisher's Note

All claims expressed in this article are solely those of the authors and do not necessarily represent those of their affiliated organizations, or those of the publisher, the editors and the reviewers. Any product that may be evaluated in this article, or claim that may be made by its manufacturer, is not guaranteed or endorsed by the publisher.
